# Advancing the Use of Longitudinal Electronic Health Records: Tutorial for Uncovering Real-World Evidence in Chronic Disease Outcomes

**DOI:** 10.2196/71873

**Published:** 2025-05-12

**Authors:** Feiqing Huang, Jue Hou, Ningxuan Zhou, Kimberly Greco, Chenyu Lin, Sara Morini Sweet, Jun Wen, Lechen Shen, Nicolas Gonzalez, Sinian Zhang, Katherine P Liao, Tianrun Cai, Zongqi Xia, Florence T Bourgeois, Tianxi Cai

**Affiliations:** 1 Department of Biostatistics Harvard T.H. Chan School of Public Health Boston, MA United States; 2 Harvard-MIT Center for Regulatory Science Harvard Medical School Boston, MA United States; 3 Division of Biostatistics and Health Data Science School of Public Health University of Minnesota Minneapolis, MN United States; 4 Department of Biomedical Informatics Harvard Medical School Boston, MA United States; 5 Department of Engineering University of Toronto Toronto, ON Canada; 6 Division of Rheumatology, Inflammation, and Immunity Brigham and Women's Hospital Boston, MA United States; 7 Department of Neurology University of Pittsburgh Pittsburgh, PA United States; 8 Computational Health Informatics Program (CHIP) Boston Children's Hospital Boston, MA United States; 9 Department of Pediatrics Harvard Medical School Boston, MA United States

**Keywords:** real-world evidence, electronic health records, chronic disease outcomes, longitudinal disease activity, machine learning, causal inference, data imputation, calibration

## Abstract

Managing chronic diseases requires ongoing monitoring of disease activity and therapeutic responses to optimize treatment plans. With the growing availability of disease-modifying therapies, it is crucial to investigate comparative effectiveness and long-term outcomes beyond those available from randomized clinical trials. We introduce a comprehensive pipeline for generating reproducible and generalizable real-world evidence on disease outcomes by leveraging electronic health record data. The pipeline first generates scalable disease outcomes by linking electronic health record data with registry data containing a small sample of labeled outcomes. It then applies causal analysis using these scalable outcomes to evaluate therapies for chronic diseases. The implementation of the pipeline is illustrated in a case study based on multiple sclerosis. Our approach addresses challenges in real-world evidence generation for disease activity of chronic conditions, specifically the lack of direct observations on key outcomes and biases arising from imperfect or incomplete data. We present advanced machine learning techniques such as semisupervised and ensemble methods to impute missing outcome data, further incorporating steps for calibrated causal analyses and bias correction.

## Introduction

Managing chronic diseases requires continuous assessment of disease activity and therapeutic response to optimize clinical treatment plans. As the availability of disease-modifying therapies (DMTs) increases, novel approaches are essential to assess comparative effectiveness and monitor long-term outcomes of these agents beyond randomized clinical trial (RCT) evidence. Real-world data (RWD) provide a comprehensive array of longitudinal clinical information from diverse patient populations, which is often challenging to gather through traditional RCTs. Consequently, real-world evidence (RWE) derived from reliable and relevant RWD can help address questions regarding the long-term effects and safety of these treatments. While disease registries are useful data sources to support RWE studies, they tend to be limited in sample size and duration of follow-up. Electronic health record (EHR) data have recently emerged as vital sources of RWD, offering invaluable insights for capturing longitudinal treatment responses in diverse patient populations and real-world settings, thus enabling the generation of RWE.

Nonetheless, the use of EHR data comes with significant challenges related to data quality and statistical analysis in the presence of imperfect data. In particular, generating RWE for DMTs with EHR data is often hindered by the lack of precise and structured data on disease outcomes, such as measures of symptom severity, functional status, relapse rates, or changes in biomarkers. These clinical measures, which are monitored by physicians during therapy, are critical for assessing treatment effectiveness but are often inconsistently recorded or embedded in unstructured text within EHRs. When valuable details on disease outcomes are captured in unstructured text fields within EHRs, they can be difficult to extract and standardize. Apart from disease outcome, the absence of critical structured data related to clinical trial inclusion criteria, treatment indications, and interventions also hinders the generation of reliable RWE from RWD. Inconsistent documentation across clinical encounters further compromises data quality, even when significant effort is invested in manual abstraction. To address these challenges and create RWE resources capable of supporting robust postmarket assessments of DMTs, it is essential to develop reproducible, scalable methods that can adapt to variations across health care systems.

Existing studies have considered the aggregation of unstructured data, the consolidation of different data modalities, and the use of new technologies to help produce more reliable RWE. For instance, Hou et al [1] emulated the Clinical Outcomes of Surgical Therapy Study Group Trial by integrating both EHR data and unstructured textual information from clinical notes. They found that with appropriate adjustment of temporal shift, RWE can supplement RCTs in the assessment of treatment outcomes over time. Patorno et al [2] further augmented EHR and insurance claims data with wearable devices and patient-reported outcomes in their treatment outcome analysis. Meanwhile, Greenbaum [3] explored integrating digital twin technology to generate RWE to support special legal programs that provided patients with access to experimental or unapproved medications. These studies, along with others [4,5], underscored ongoing challenges in obtaining accurate and reliable disease outcomes, and emphasized how new technologies and methodologies can help address these limitations.

Advanced imputation techniques are essential for producing more accurate and reliable disease outcomes [3]. While a range of supervised algorithms such as random forest, logistic regression, support vector machines, and various deep learning models can be used to impute outcomes, these methods may not be effective when the number of gold-standard labels, generated either from manual chart review or from EHR-linked disease registries, remains modest. Further, imputed outcomes regardless of the imputation methods are probabilistic in nature, and the direct use of such imperfect outcomes in downstream causal modeling can introduce biases in the estimated treatment effect. The existing literature lacks a systematic pipeline for producing high-quality, label-efficient imputations, along with calibrated causal modeling methods that can generate reliable RWE to complement RCT data.

**Figure 1:**
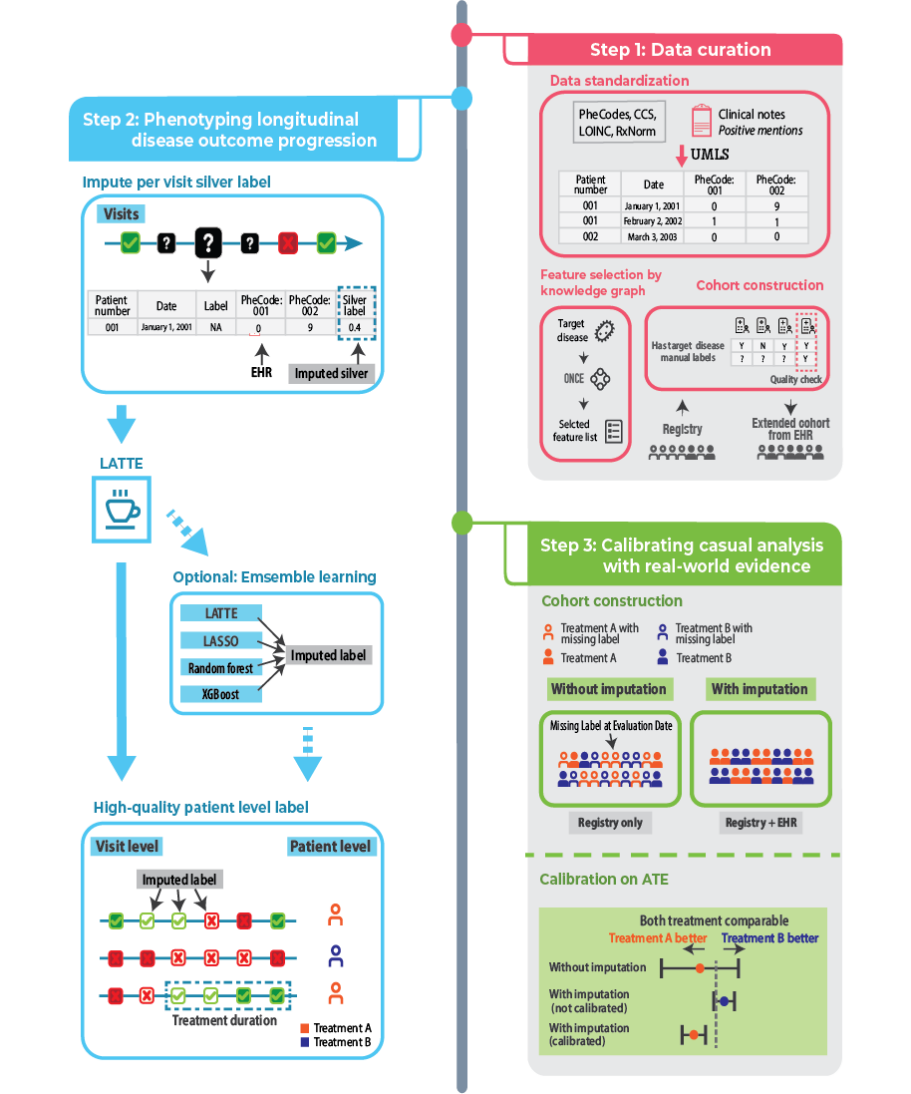
Diagram of the full pipeline. ATE: average treatment effect; CCS: Clinical Classifications Software; EHR: electronic health record; LASSO: least absolute shrinkage and selection operator; LATTE: label-efficient incident phenotyping; LOINC: Logical Observation Identifiers Names and Codes; RxNorm: Normalized Naming System for Generic and Branded Drugs; UMLS: Unified Medical Language System.

To advance the use of RWE as a complement to RCTs, we present a tutorial on a pipeline designed to produce reproducible and generalizable RWE on disease outcomes, specifically for evaluating therapies in chronic diseases, using multiple sclerosis (MS) as a demonstration case. Our pipeline, illustrated in Figure 1, generates scalable disease outcomes by linking EHR features with disease outcome data from registries and developing algorithms based on EHR-derived features. To mitigate potential biases in causal effect estimates associated with imperfect outcomes generated from imputation, we outline a calibrated causal modeling estimation method. The pipeline builds on existing tools developed in the literature [6,7], with a unique focus on generating reliable longitudinal disease outcomes.

## Methods

### Pipeline Overview

Our pipeline contains three key steps: (1) data curation, (2) phenotyping of longitudinal disease outcomes, and (3) calibration of causal analysis with RWE. For reference, in Table 1, we provide a comprehensive list of terms and their definitions used throughout the tutorial.

**Table 1:** Definitions of terms.

Term	Definition
Codified features	Including diagnosis codes (grouped by PheWAS^a^ catalog and PheCode), procedure codes (grouped by CCS^b^), medication codes (grouped by RxNorm^c^), and other code systems (eg, laboratory tests grouped by LOINC^d^).
NLP^e^ features	Clinical terms mentioned in narrative notes grouped by medical concepts from UMLS^f^.
Embedding	Numeric vector representations of features [8] that capture the relationships between the features. Specifically, related features tend to have embeddings that are closer together in the vector space (eg, higher cosine similarity), while unrelated features are farther apart.
Knowledge graph	Structured representation that captures the pairwise relationship among clinical features.
Clinical outcome, outcome, or label	Used interchangeably with “disease outcome,” referring to the outcome of interest in the RWE^g^ study.
Health care use	Measure of volumes of health care encounters for each patient, reflecting overall health and noise level in data. Typically calculated by total days with any codified features.
Baseline confounders	Features that may associate with both the treatment and the outcome of a study, which would distort the observed treatment-outcome association.
Gold-standard labels	Clinical features annotated by clinical experts through manual EHR^h^ review or available from research registries.
Silver-standard labels	Computable but noisy proxies of gold-standard labels that can be readily extracted for all patients. Used interchangeably with “surrogate outcome.”
Labeled patient	Patients with gold-standard labels.
Unlabeled patient	Patients without gold-standard labels.
Patient period	Patient’s longitudinal history divided into equal-length intervals, based on the typical frequency of clinical assessments.
Labeled patient period	Patient period with gold-standard label.
Unlabeled patient period	Patient period without gold-standard label.
Scalable	Applicable to large study cohorts with focus on (1) requiring few gold-standard labels for training, and (2) deployable to another research environment.
Semisupervised finetuning	Process whereby the pretrained model is further trained on both the labeled and unlabeled datasets, allowing refinement of predictions.
Unsupervised pretraining	Process whereby a model is initially trained on a large, unlabeled dataset to learn general features and patterns.

^a^PheWAS: Phenome-Wide Association Study.

^b^CCS: Clinical Classifications Software.

^c^RxNorm: Normalized Naming System for Generic and Branded Drugs.

^d^LOINC: Logical Observation Identifiers Names and Codes.

^e^NLP: natural language processing.

^f^UMLS: Unified Medical Language System.

^g^RWE: real-world evidence.

^h^EHR: electronic health record.

#### Use Case

In this tutorial, we use a case study based on MS to illustrate the deployment of our pipeline in conducting an observational clinical research study. The clinical question of interest is to compare the long-term treatment effectiveness between 2 groups of high-efficacy MS DMTs, namely, B-cell depletion therapies (eg, ocrelizumab) versus alpha4-integrin blockers (eg, natalizumab). For MS, a common treatment efficacy measure in RCTs is the reduction in acute inflammatory relapse events. A relapse event is defined either clinically, based on persistent neurological symptoms lasting for more than 24 hours, or radiologically, based on new or enlarged white matter lesions on magnetic resonance imaging (MRI) [9]. It is coded as a binary disease outcome, with “1” indicating a relapse event and “0” indicating no relapse event. Routine clinical encounter notes and MRI reports often contain information informative of relapse occurrence, but the heterogeneity in clinical practice and documentation patterns in routine clinical settings impose challenges to the scalable extraction of relapse data. To obtain a set of patients with gold-standard labels, we used data from a clinic-based research registry (ie, the Prospective Investigation of MS in the Three Rivers Region), which is linked to the University of Pittsburgh Medical Center EHR system. This tutorial provides a step-by-step guide on enhancing the detection and extraction of valuable information from EHR, applying computational methods that leverage a few gold-standard labels to accurately impute the clinical outcomes, and using calibration steps to ensure robust estimation of the average treatment effect, illustrated through the MS case study.

### Data Curation

#### Data Standardization

To analyze the target clinical outcome, the first step is to create an EHR data mart that includes both structured (diagnosis codes, procedures, medications, and laboratory tests) and unstructured data (clinical notes). Clinical outcomes, often embedded in notes but lacking specific codes (eg, relapse), necessitate the inclusion of both types of data. Codified (structured) data are grouped into standardized formats using ontologies like Phenome-Wide Association Study catalog (PheCode), Clinical Classification Software, Normalized Naming System for Generic and Branded Drugs (RxNorm), and Logical Observation Identifiers Names and Codes [10-12]. Narrative (unstructured) data are processed using natural language processing (NLP) tools (eg, NILE [13], MetaMap [14], cTAKES [15]), which identify and map mentions of clinical terms to concept unique identifiers. See Hou et al [6] and Hong et al [16] for details on leveraging ontologies to group EHR features.

#### Feature Selection by Knowledge Graph

**Figure 2:**
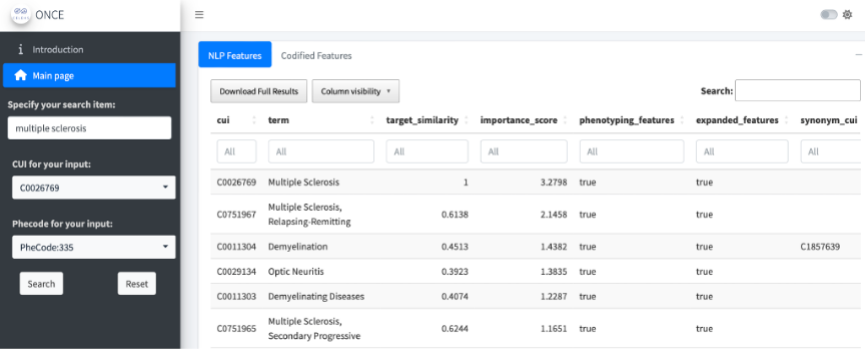
Illustration of ONCE Shiny app. Users can input the names of the disease of interest at “Specify your search item” and download the feature dictionaries by clicking “Download Full Results” under “NLP Features” and “Codified Features.” NLP: natural language processing.

Given the vast number of features (both structured and unstructured) present in EHRs, implementing an effective filtering strategy to focus on a subset of clinically informative features for downstream imputation and causal modeling is essential to avoid overfitting and improve generalizability. Manual selection of informative features by clinical experts, while useful in maintaining interpretability, is prone to subjective bias and lacks scalability. In addition, manual curation requires an additional step of mapping clinical features of interest to EHR codes and NLP concepts, creating additional sources of subjective bias and error. A scalable and generalizable approach to automated feature selection is to leverage comprehensive knowledge graphs for EHR concepts, which encode a degree of relatedness among these concepts. Existing knowledge graphs, for example, KESER, ARCH, ONCE, Code2Vec, and Med2Vec, derived from EHR data, can be used to identify features important for specific diseases, treatment, and target clinical outcomes [16-20]. The knowledge graphs mentioned here are primarily constructed using co-occurrence matrices of EHR concepts and their textual descriptions, and cosine similarities between concepts are used to measure the strength of pairwise relatedness. These tools enable the identification of relevant EHR codes and NLP-derived narrative concepts for a given clinical condition of interest. For instance, key concepts for MS may include International Classification of Diseases (ICD) codes for the condition, narrative mentions of MS, MS-specific medication prescriptions, MRI procedures, and narrative mentions of brain lesions. Xiong et al’s [17] knowledge graph offers a practical resource for assembling disease-specific features through a Shiny app web service called ONCE, illustrated in Figure 2. To assess the quality of knowledge graphs for a user’s specific disease of interest, a curated set of entity pairs with known similarity or relatedness can be used. The effectiveness of the knowledge graphs can then be evaluated by determining whether the cosine similarities they generate can distinguish these known pairs from unrelated ones. More details on this process can be found in a study by Gan et al [20].

It is important to recognize that some clinical features are inadequately represented by specific structured codes and cannot be easily mapped to structured data. Hence, using purely knowledge graph–based automated feature selection can lead to the omission of clinically important features that are underrepresented in the graph. To address this limitation, we recommended expanding the mapping process to include some other key relevant features, suggested by clinical experts but not considered or selected by the automatic feature selection procedure previously. While this hybrid approach may introduce some redundancy, our pipeline includes data-driven selection mechanisms in both the phenotyping step (eg, the concept reweighting [CR] module) and the causal calibration step (eg, penalized propensity score and outcome regression models), which effectively down-weight or exclude noninformative features, ensuring that their inclusion does not negatively impact performance.

#### Cohort Construction

To identify the target disease cohort, a crucial step is to accurately classify the disease status at the patient level. While ICD codes tend to be reasonably sensitive in capturing patients with the disease, they often lack specificity [21-23]. Ascertaining the disease status from EHR has been studied as a phenotyping problem [7]. Weakly supervised phenotyping algorithms such as KOMAP, PheCAP, Anchor, APHRODITE, PheNorm, and MAP are more accurate alternatives to ICD-based classification [17,24-28]. These algorithms typically use surrogate features as “noisy labels” to supervise the training of models that integrate additional EHR data for more accurate disease classification. Key surrogate features include relevant ICD codes, narrative mentions of disease terms, and health care use metrics. Algorithms provide likelihood scores, allowing researchers to set specificity thresholds for classifying disease presence. These weakly supervised algorithms are recommended due to their ability to train without gold-standard labels.

#### Use Case

To examine the treatment effect, we constructed an MS EHR data mart containing codified and narrative EHR features for patients meeting inclusion criteria. For example, patients should have key demographic information, including gender, age, race, and ethnicity, and have been in the EHR system for at least 6 months prior to their first DMT treatment. We mapped each patient’s diagnosis, laboratory, medication, and procedure codes from the local, institutional-level codes to a common ontology, and then aggregated them hierarchically. For feature selection, we searched “multiple sclerosis” using the ONCE tool, which selected MS-relevant features such as “optic neuritis” and “dimethyl fumarate.” The unsupervised MAP algorithm was used for diagnosis phenotyping, leading to a subset of patients identified as having MS. Further implementation details are available in the paper by Xiong et al [17].

### Phenotyping Longitudinal Disease Outcome

#### Overview of Semisupervised Incidence Phenotyping Methods

Semisupervised incidence phenotyping methods can be applied when longitudinal disease outcomes are incomplete due to sparse documentation or are completely missing. Specifically, these methods leverage information contained in the unlabeled data via structured exploration (eg, tensor structured factorization [29]) or the construction of silver-standard labels [24-26]. Unlike the previously discussed weakly supervised algorithms, however, these methods require a small sample of gold-standard labels to guide model training. Among these approaches, the label-efficient incident phenotyping (LATTE [30]) method stands out for its requirement for minimal gold-standard labels (ie, label efficiency) and integration of knowledge graphs, effectively enabling outcome phenotyping in a data-sparse environment.

#### Data Preparation

To phenotype longitudinal disease outcomes, the first step is to define the time window during which the EHR data are aggregated and the outcome is ascertained, referred to as the “patient period.” The outcome represents an “average” outcome during the time window for each patient. For each patient period, three types of data need to be collected: (1) gold-standard labels representing the disease outcome, (2) selected and aggregated EHR codified and narrative features, and (3) constructed silver-standard labels, which serve as proxies for the gold-standard labels. The curation steps are as follows.

- Obtaining gold-standard labels: Gold-standard labels are critical for accurate modeling. Longitudinally collected gold-standard labels should be available in at least 100 patients to ensure robust algorithm training and validation. These labels can be sourced from EHR-linked disease registries or through careful manual chart reviews by trained researchers who follow standardized annotation protocols. For patient periods where gold-standard labels are unavailable, they should be marked as “NA” to indicate unlabeled status. A patient in the labeled set may not have all their periods labeled.
- Knowledge-guided feature selection and aggregation: Given the limited availability of gold-standard labels and the complexity of modeling longitudinal disease outcomes, knowledge-guided feature selection and aggregation are crucial. One may select relevant features using the knowledge graph along with semantic embeddings of the EHR features. The embedding vectors can be obtained from pre-trained embeddings, such as those powering the ONCE search engine, or by embedding the textual descriptions of the features using large language models such as Clinical BioBERT [31], CODER [32], BGE [33], or even GPT-4 [34]. The selected features can be optionally reviewed and further augmented by clinical experts to include additional informative features.
- Constructing silver-standard labels: LATTE’s label-efficient approach uses predictive silver-standard labels to effectively train models on large, unlabeled datasets. These silver-standard labels for each patient period can be generated through different methods depending on the target outcome. For outcomes that can be effectively captured by diagnostic codes, one may aggregate the diagnosis codes most relevant to the outcome of interest. For outcomes that are well described in clinical notes, one may use advanced NLP tools to extract relevant terms. Alternatively, if disease activity correlates with changes in monitoring or treatment strategies, one may use specific EHR features—such as diagnoses or treatments—as proxies for adverse outcomes. For more complex outcomes, such as disability or remission, a data-driven approach may be required to combine several candidates’ features to form a surrogate outcome using standard supervised training with a small number of labels as a pretraining step. The predicted probabilities generated from this pretraining can serve as the silver-standard labels.
- Organizing the input data: The data components must then be organized and formatted for analysis. This involves structuring the data into files compatible with the LATTE framework, including training and testing datasets, feature lists, and embedding files. The input data should maintain a consistent format across all patient periods, with missing data appropriately flagged. Detailed examples of input data formats and file structures are provided on our webpage in
  Multimedia Appendix 1
  to facilitate seamless integration into the LATTE pipeline.

#### Training the LATTE

**Figure 3:**
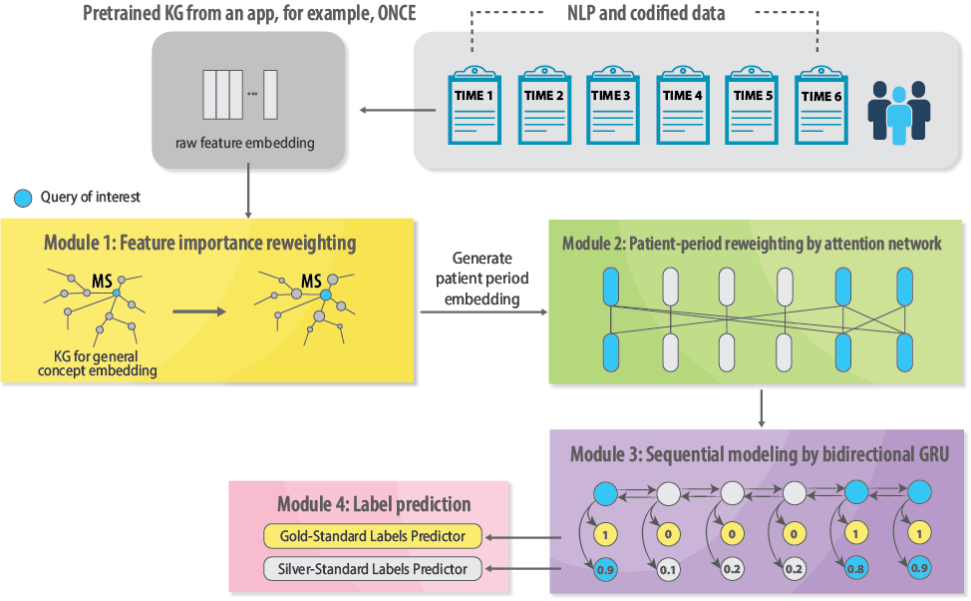
Illustration of LATTE workflow. GRU: gated recurrent unit; KG: knowledge graph; LATTE: label-efficient incident phenotyping; MS: multiple sclerosis; NLP: natural language processing.

Figure 3 illustrates the workflow of LATTE, which processes 3 key types of input data. The first is a longitudinal data frame indexed by patient periods. This data frame includes numeric columns for aggregated counts of selected EHR features, numeric columns for demographic or clinical features, a numeric column for gold-standard labels, a numeric column for silver-standard labels, and a Boolean column indicating the presence or absence of gold-standard labels. The second input consists of prederived embedding vectors for the selected EHR features, which encode the relationships between these features as a knowledge graph. The third input is the “query of interest,” which refers to a specific EHR feature among the list of selected features identified as highly predictive of the disease outcome of interest. For instance, if the outcome of interest is relapse, the procedure code for an MRI scan might be selected as the “query of interest” due to the strong association of this procedure with relapse events.

Input data are divided into training and validation sets. LATTE processes the trained data through 4 computational modules using a 2-stage training strategy.

- CR: The CR module assigns weights to each input feature based on its semantic relevance to the clinical outcome, thereby mitigating feature collinearity and reducing the risk of overfitting. For example, when phenotyping longitudinal MS relapse, the procedure code for an MRI scan, encoded as “CCS:198,” is highly indicative of relapse events. Therefore, it will be used as the “query of interest” and the CR module will assign higher weights to features semantically related to this code, such as optic neuritis, while reducing the weights of less relevant features, such as mental health conditions. Specifically, a multilayer perceptron network within the CR module is trained to adjust feature importance by amplifying the weights of related features and diminishing the weights of less relevant ones. Unlike methods that rely solely on embedding similarity, the CR module dynamically accounts for feature prediction, even when semantic similarity does not directly correlate with predictive accuracy.
- Patient periods attention network (PPAN): The PPAN module is designed to assign greater importance to patient periods that provide significant information about disease progression, thereby minimizing the impact of noninformative periods. For example, a patient with MS may have several patient periods informative of their MS care and outcome, but there may also be periods of less direct relevance to MS, such as those pertaining to treatment for influenza infection or management of other chronic conditions such as diabetes. By using a self-attention layer, PPAN effectively highlights the most informative patient periods within a sequence, thus enhancing the model’s capability to capture long-range temporal dependencies and improve outcome predictions.
- Sequential modeling with the bidirectional gated recurrent unit (bi-GRU): LATTE uses a bi-GRU to model temporal dependencies between patient periods. This approach integrates both past and future patient period information when predicting incident events as phenotypes. The bi-GRU processes patient period embeddings from the CR module, along with attention values from the PPAN, while incorporating numerical demographic or clinical features, to generate patient period representations.
- Label prediction: Two separate loss functions are combined to predict the gold- and silver-standard labels.
- Unsupervised training stage with silver-standard labels: LATTE first undergoes unsupervised training using silver-standard labels generated from the previous step. The total number of iterations for the pretraining phase requires specification in the algorithm. We recommend setting a large number of iterations, for example, more than 60, if the silver-standard labels are of high quality. Otherwise, it should be set to a small number of iterations, for example, less than 20. The pretrained model parameters should be saved to a prespecified output directory and then loaded during the fine-tuning phase.
- Semisupervised training stage with gold- and silver-standard labels: After initializing with the pretrained model parameters, LATTE undergoes semisupervised training using both gold- and silver-standard labels. Separate loss functions for each label type prevent the potential noise related to silver-standard labels from degrading the model’s learning from gold-standard labels. Specifically, based on the quality of the silver-standard labels, an additional hyperparameter “unlabel_weight”, which lies between 0 and 1, can be used to control the contribution of the training with silver-standard labels. We suggest setting the weight to a value less than 0.5 to ensure efficient learning by models from high-quality gold-standard labels.

To validate the performance of the trained LATTE model, it is further used to impute the disease outcome on the held-out validation set, which is then benchmarked against the gold-standard labels in the validation set. Currently, LATTE only supports the prediction of binary or time-to-event disease outcomes, and its outputs are imputed probabilities between 0 and 1. Currently, LATTE only supports the prediction of binary or time-to-event disease outcomes, and its outputs are imputed probabilities between 0 and 1. For time-to-event outcomes, the event timing data can be first converted into a sequence of 0s and 1s, indicating whether the event has occurred at regular time intervals. An example of time-to-heart failure was previously provided [30]. For other types of outcomes such as continuous outcomes, other existing methods may be adopted instead [35].

Since training LATTE is the most computationally and time-intensive step in our pipeline, we provide empirical benchmarks to illustrate the resource requirements based on our implementation. Specifically, for a dataset with 100 features**,** 5000 EHR patients with 100,000 unlabeled patient-by-time records, and 500 registry patients with 3000 labeled records, the LATTE algorithm takes approximately 2 hours to train on a single-core CPU with 80 GB of memory. When the size of the feature set is increased to 300 (with the same number of patients and labels), the training time increases to approximately 4 hours on a single-core CPU with 120 GB of memory. For significantly larger datasets, we recommend a divide-and-conquer strategy and leveraging GPU resources if available. Specifically, unlabeled data can be split into batches, with each batch paired with the labeled subset during training. Each batch would produce one prediction model, and the final prediction for a future patient would be the average of the predicted probabilities from different batches. Such a strategy allows the method to scale more efficiently while keeping memory usage manageable.

#### Ensemble Learning

To enhance prediction accuracy, LATTE-imputed disease outcomes can be combined with those derived from other machine learning models. Traditional supervised or semisupervised methods, such as least absolute shrinkage and selection operator (LASSO), random forest, and extreme gradient boosting, can be used to develop imputation models that predict the outcomes based on the selected EHR features as well as demographic or clinical features.

To improve robustness and reduce the risk of overfitting, we recommend aggregating the predictions from multiple models using a simple cross-fitted approach and refer to this process as “ensemble learning”. The process involves the following.

- Data splitting: Divide the labeled data in the training set into multiple folds.
- Cross-fitting: For each fold, train LATTE and other machine learning models with out-of-fold data and generate predicted disease outcomes for the in-fold patient periods. In particular, LATTE can use the full unlabeled data in each cross-fitting step. After iterating through all folds, the predicted outcomes are obtained for the entire training set.
- Model aggregation: Once predicted disease outcomes are obtained from all models, a simple supervised parametric model is trained to combine them and produce a final ensemble model.
- Model validation: The ensemble model is used to produce predicted outcomes on the validation set, which are then benchmarked against the gold-standard labels in the validation set.

This ensemble method leverages the complementary strengths of various algorithms, leading to enhanced prediction performance.

***Use Case***

For the MS case study, the disease outcome was the 3-month relapse status (0=no relapse and 1=has relapse). In clinical practice, relapse events are typically documented as they are discovered (eg, a clinical episode, or a neuroimaging or MRI finding) and at a frequency or interval shorter than routine clinical visits (whether once per 6 months or 1 year). Thus, we chose to phenotype relapse at 3-month intervals. A set of EHR features was selected using ONCE and augmented with an additional set of EHR features (eg*,* medications, radiological findings) as suggested by clinician experts. For all patients, the patient history period spans from the date of the first MS ICD code to the date of the last documented visit. This period was then divided into consecutive 3-month patient periods. For each patient period, we obtained the aggregated counts of each feature for each patient. Since relapse events were only documented at specific visit dates, we further aggregated the labels across the entire 3-month period to obtain the gold-standard labels for each patient period.

To create silver-standard labels, we trained a logistic regression model on a sample of 100 labeled patients using radiology-related features suggested by a clinician expert. We leveraged the knowledge graph from the ONCE search engine. Given that we were interested in imputing relapse, the query of interest was set to the procedure code for a relevant type of MRI scan (eg, brain MRI). The labeled dataset was divided into training and validation sets, and we further used a set of data from unlabeled patients. The ability to leverage the unlabeled patients boosts the accuracy of LATTE. On the validation set, the LATTE model outperformed other methods, achieving an area under the curve of 0.7897, compared to LASSO at 0.7295, the random forest at 0.7566, and XGBoost at 0.7368. An ensemble with these four methods reached an area under the curve of 0.8072, demonstrating its advantage.

### Calibrating Causal Analysis With RWE

#### Deriving Baseline Covariates and Disease Outcome from EHR Data

A causal analysis requires prespecification of the following key components: eligibility criteria, treatment arm selection, period of evaluation, and list of potential baseline confounders. The list of potential baseline confounders could contain both clinician-suggested features and ONCE-selected features. However, certain important baseline confounders, such as baseline disease outcomes, may still be missing from the EHR and require imputation before causal analysis.

In the context of observational studies where only a small subset of patients would have gold-standard disease outcome labels, the imputed outcome from the aforementioned algorithms would be critical to ensure that the study has sufficient power. We recommend using the ensemble approach to impute the outcome for the downstream causal analyses.

#### Calibrating Outcomes for Unbiased Average Treatment Effect Estimation

Imputation accuracy alone cannot guarantee small estimation bias in the causal analysis of average treatment effect (ATE) using imputed outcomes. Disproportionate imputation error across treatment groups, for example, underestimated outcomes for 1 treatment and overestimated outcomes in the other, may introduce bias even if the imputation has demonstrated high classification accuracy [36]. Moreover, machine learning–based imputations sometimes can only approximate the true outcomes to a certain degree. The discrepancies between imputed and true outcomes can further compromise the accuracy of ATE estimates, leading to potentially erroneous conclusions. Therefore, it is crucial to leverage gold-standard outcomes for assessing the bias from imputed outcomes and correct such bias through the calibration of imputed outcomes as part of the ATE estimation process.

Several existing calibration methods for causal analysis using imperfect outcomes [37,38] with or without other imperfect data components [36] all fundamentally follow a similar strategy: first calibrating the imputation of outcomes, and then estimating ATE according to annotated outcomes over the labeled subset. To reduce heterogeneity in imputation error across treatment groups or different values of key confounders, a calibrated imputation model incorporating imputed outcomes, treatment, and confounding features is trained using annotated outcomes. Integrating treatment and confounding features in the calibration model ensures minimal correlation between imputation error and these key features [39]. The remaining bias in calibrated imputation for ATE is then estimated over the labeled subset using annotated outcomes and corrected using the augmentation of inverse probability weighted residual.

We introduce the following notations: Y denotes the true disease outcome, and A is the treatment assignment indicator, where A=1 indicates treatment and A=0 indicates control or comparator.

The algorithm for calibrated estimation of ATE involves the following steps [36,38].

- Fitting the propensity score (PS) model and outcome regression (OR) model: Based on the standard doubly robust estimation of ATE [40,41], our calibrated estimation also involves modeling the PS and OR models. PS is the probability for a patient to receive a specific treatment indicated by A=1, given their observed baseline characteristics. It can be estimated by regressing the observed treatment assignment A on the baseline characteristics. For instance, in our MS case study, we regressed the treatment indicator (A=0 for natalizumab; A=1 for B cell depletion) on the baseline characteristics (eg, baseline demographics, baseline disability status). The estimated PS is then used to weigh the observed treatment arms such that each has a similar distribution of baseline characteristics to the full study cohort after weighting. The OR model, on the other hand, predicts the potential outcomes following 2 competing treatments based on baseline characteristics. Since gold-standard outcomes are only available for a labeled subset of patients, the OR model is estimated by regressing the observed true disease outcomes on the treatment indicators, baseline characteristics, and their interactions. In our MS case study, for instance, we regressed the disability outcome (Y=0 for mild to moderate disability and Y=1 for severe disability) on the treatment indicators and baseline confounders.
- Calibration of the imputed outcomes: The gold-standard outcomes are used again to reduce the association between imputation error and other key factors (treatment and confounding). Specifically, calibration is performed by regressing the available true disease outcomes on treatment indicators, baseline confounders, and preliminary imputed outcomes obtained through the ensemble method. The predicted values from this model are referred to as the “further calibrated outcomes.”
- Calibrated estimation of ATE: Over the labeled subset of patients, we estimate the remaining bias from the further calibrated outcomes by contrasting ATE based on the observed outcomes and ATE based on the further calibrated outcomes. The final calibrated estimation of ATE is then obtained by correcting the ATE calculated using the further calibrated outcomes across the full patient cohort by the estimated bias.

Regressions can be alternatively estimated via machine learning methods. Cross-fitting is recommended to reduce overfitting bias [36]. We highlight the method by Kallus and Mao [38] as it addresses the challenge of selecting a small set of confounding features from numerous potential candidates, which is common in RWE studies.

#### Use Case

To conduct a causal analysis of the effect of MS treatments on reducing relapse events, we compared two commonly prescribed classes of high-efficacy DMTs: natalizumab versus B cell depletion, which includes ocrelizumab and ofatumumab. Natalizumab was assigned as the control or active comparator group (ie, A=0) and the latter as the treatment (of interest) group (ie, A=1). Patients were categorized into either the natalizumab group or the B cell depletion group based on the first treatment recorded in the data. The period of outcome ascertainment was set to 1 year after the start of treatment. The disease outcome Y was a 1-year MS relapse status, which is defined by whether a patient experienced any relapse within a year. The imputed outcomes were derived using the previously described methods. Specifically, we first derived the 3-month relapse probability and then applied a rolling aggregation to convert every 4 consecutive 3-month relapse probabilities into a 1-year relapse probability. To address potential confounding, we included several baseline covariates. These covariates consisted of expert-defined demographic and clinical features, such as age, gender, race and ethnicity, disease duration based on the time from MS diagnosis to treatment initiation, and the imputed baseline relapse status 1 year prior to the start of treatment. We also included the log counts of ONCE-selected EHR features relevant to MS aggregated over the 1-year period prior to the start of treatment as additional covariates.

We have applied both the method proposed by Kallus and Mao [38] and Cheng et al [37] to calibrate the imputed outcomes and obtain a robust estimate of ATE. The second method differs slightly from the previous description and is summarized below for the reader’s reference. The first step involved fitting a “double-index” propensity score model. Both the initial PS model and the initial outcome regression were fitted using adaptive LASSO regression. We then used a fourth-order kernel for smoothing in conjunction with a local constant regression approach to refine the double-index PS model. In addition to the imputed outcome, we included the log counts of ONCE-selected EHR features aggregated after the start of treatment as additional covariates. The calibration model was then fitted using adaptive LASSO regression, and the further calibrated outcome was subsequently used to estimate ATE. Further details are provided in Multimedia Appendices 1 and 2.

## Discussion

### Summary

This tutorial presents a comprehensive pipeline for generating reproducible and generalizable RWE on disease outcomes using EHR data. Our approach addresses key challenges, such as incomplete data and bias, through advanced longitudinal imputation and calibration techniques. We demonstrate the pipeline using MS as a use case, highlighting its practical application in clinical research.

### Limitations

The proposed pipeline has several limitations that could impact the reliability and validity of findings in studies using RWD. Chief among them, the accuracy of the semisupervised phenotyping algorithm heavily depends on the quality of silver-standard labels. Although advanced phenotyping algorithms and NLP techniques are used to generate silver-standard labels, inconsistent definitions, and documentation practices across various EHR systems may compromise their quality. This inconsistency is especially problematic in areas such as cancer progression and recurrence, where essential metrics are either unmeasured or inconsistently documented, posing a risk to the reliability of silver-standard labels and subsequently the generalizability of our phenotyping algorithm.

Second, the reliability of the causal analysis step in the proposed pipeline relies on appropriately adjusting the standard methods to the study of interest as well as the specific features of the data. Missing outcomes in EHR are often informative rather than random, which can introduce potential bias into analyses. Specialized weighting approaches in causal analysis are employed to mitigate this issue, though these methods cannot guarantee the elimination of all biases. The absence of critical features limits the depth and accuracy of analyses. Variability in baseline disease outcomes affects both treatment selection and subsequent outcomes, requiring careful consideration in study design and interpretation. Data source variability, particularly concerning covariate shifts between labels derived from registries and those obtained from EHR data, may challenge the generalizability of findings from one cohort to another.

Third, handling long-term outcomes introduces additional complexity due to treatment modifications post baseline and time-varying confounders, such as changes in patient behaviors that influence both treatment and outcomes. Temporal shifts, including changes in treatment practices or patient demographics over time, further complicate the distinction between treatment effects and time effects.

Finally, the observational nature of studies using RWD inherently includes confounding factors that routine adjustment methods may not adequately address, leading to potentially biased causal inferences. The presence of unmeasured confounders, which could influence both the treatment and the outcome, exacerbates this risk. While we can estimate the unmeasured confounding in relation to the expected treatment effect, the choice of calibration models is critical and must be made with careful consideration to ensure the validity of study conclusions.

Potential solutions to address the aforementioned limitations include the following strategies.

- Enhancing data standardization. This can be achieved through 3 steps. First, the adoption of structured templates such as STaRT-RWE [9] ensures that essential study details—such as inclusion or exclusion criteria, definitions of treatments and outcomes, and handling of missing data—are consistently documented. This, in turn, facilitates the appropriate selection of analytical techniques. Second, leveraging artificial intelligence (AI) agents for unstructured data processing. Large language model–based AI systems can play a transformative role by extracting structured information from clinical notes. These models can identify medical entities (eg, diagnoses, medications, symptoms), determine their assertion status (eg, present, absent, possible), extract numeric values from lab reports, and capture temporal details such as onset and duration. Once extracted, this information can be reformatted into standardized templates, supporting downstream phenotyping and analytical workflows [42]. Third, developing cross-institutional ontologies and terminology mappings. Given the heterogeneity across EHR systems, unified terminology mapping tools [17] can help align variable definitions across institutions and reduce inconsistencies.
- Improving the generalizability of data imputation and causal analysis: First, data projection and harmonization techniques—such as canonical correlation analysis, manifold alignment, or autoencoder-based representation learning—can be used to project datasets from multiple sources into a shared latent space, mitigating covariate shifts and improving model transferability across cohorts [43,44]. Second, federated learning approaches enable collaborative model training across institutions without the need to share raw data, thus preserving privacy while enhancing generalizability. Third, building on federated representation learning frameworks, federated causal inference methods allow for robust estimation of treatment effects across sites [45]. Adaptive approaches within this framework can dynamically adjust to site-specific data characteristics and distributions.
- Incorporating structural and temporal characteristics of the study cohort into the analysis pipeline: First, in long-term observational studies, incorporating calendar time as a confounding variable can help address temporal shifts due to evolving clinical practices, updated guidelines, or changes in patient demographics. This can be done through stratification or by including calendar time as a covariate or spline in causal models [1]. Second, when uncertainties exist about the temporal behavior of covariates or treatment effects, simulation-based sensitivity analyses can be used to assess the robustness of the findings under a range of assumptions.
- Reducing confounding through instrumental variables (IVs): IV analysis can help isolate exogenous variation in treatment assignment when confounding is suspected. Identifying valid IVs can be supported through domain expertise and recent machine learning–based techniques for IV discovery and validation.
- Leveraging external data sources: Complementary data sources such as disease registries, genomic databases, claims data, or patient-reported outcomes can enhance EHR-derived datasets by improving feature completeness and labels for training phenotyping algorithms. Integration of these sources—through multimodal learning—can also strengthen causal inference.

As with all EHR studies, careful consideration must be given to potential ethical issues. Institutional review board approval is typically required prior to accessing and analyzing EHR data. When connecting EHRs with registry data, appropriate consent procedures and data use agreements should be established. Researchers must also implement safeguards to protect patient confidentiality and ensure compliance with applicable regulations.

### Conclusions

In conclusion, we present a pipeline for generating RWE on disease outcomes using EHR data. By leveraging advanced machine learning techniques and calibration methods, we address key challenges in RWE generation, providing a scalable and generalizable framework for evaluating therapies in chronic diseases. Despite its limitations, this pipeline marks a significant advance in the effective generation of RWE to complement RCT evidence, offering valuable insights for clinical decision-making.

## Appendix

Multimedia Appendix 1 Website.

Multimedia Appendix 2 Mathematical details for causal calibration step.

## Abbreviations

AI: artificial intelligence
ATE: average treatment effect
Bi-GRU: bidirectional gated recurrent unit
CR: concept reweighting
DMT: disease-modifying therapy
EHR: electronic health record
FDA: US Food and Drug Administration
HHS: Department of Health and Human Services
ICD: International Classification of Diseases
IV: instrumental variable
LATTE: label-efficient incident phenotyping
LASSO: least absolute shrinkage and selection operator
MRI: magnetic resonance imaging
MS: multiple sclerosis
NLP: natural language processing
OR: outcome regression
PPAN: patient periods attention network
PS: propensity score
RCT: randomized clinical trial
RWD: real-world data
RWE: real-world evidence
RxNorm: Normalized Naming System for Generic and Branded Drugs
