# Supplementary material for: Advancing the Use of Longitudinal Electronic Health Records: Tutorial for Uncovering Real-World Evidence in Chronic Disease Outcomes
Source: J Med Internet Res. 2025 May 12;27:e71873. doi: 10.2196/71873 (PMC12107207; doi:10.2196/71873)
Supplement: Multimedia Appendix 1 [file jmir_v27i1e71873_app1.docx]

**Multimedia Appendix 1. Website**

We provide a website with example codes for readers who are interested to implement the methods proposed in this tutorial.

<https://celehs.github.io/rwe-tutorial/>
