# Supplementary material for: Advancing the Use of Longitudinal Electronic Health Records: Tutorial for Uncovering Real-World Evidence in Chronic Disease Outcomes
Source: J Med Internet Res. 2025 May 12;27:e71873. doi: 10.2196/71873 (PMC12107207; doi:10.2196/71873)
Supplement: Multimedia Appendix 2 [file jmir_v27i1e71873_app2.docx]

**Multimedia Appendix 2. Mathematical details for causal calibration step**

This appendix includes the mathematical details in Kallus and Mao [33] and Cheng et al. [32]. Firstly, we introduce the following notations: Y – true disease outcome, A – treatment assignment indicator (A=1 for treatment and A=0 for control or comparator), R – observation indicator (R=1 for labeled and R=0 for unlabeled), S – imputed outcome obtained through the ensemble method discussed previously, X – baseline confounders, and S′ – outcome further calibrated.

The algorithm for calibrated estimation of ATE in Kallus and Mao [33] involves the following steps:

1. **Fitting the propensity score (PS) model and outcome regression (OR) model.** Based on the standard doubly robust estimation of ATE [53,54], our calibrated estimation also involves modeling the PS and OR models. The PS can be estimated by regression, $A_{i}\sim\pi(X_{i})$, using the full cohort. Since the gold-standard outcomes are only available over the labeled subset with $R_{i}=1$, the OR is estimated by regression, $Y_{i}\sim\mu(A_{i},X_{i})$.
2. **Calibration of the imputed outcomes.** The gold-standard outcomes are used again to reduce the association between imputation error and other key factors (treatment, confounding). The calibrated imputation estimated by regression, $Y_{i}\sim\eta(A_{i},X_{i}{,S}_{i})=S'_{i}$.
3. **Calibrated estimation of ATE.** Denote the doubly robust estimator formula

$$\phi(Y,A,X)=\mu(1,X)-\mu(0,X)+\frac{A}{\pi(X)}\{Y-\mu(1,X)\}-\frac{1-A}{1-\pi(X)}\{Y-\mu(0,X)\}$$

for contribution of individual observation with outcome Y, treatment T, and confounder X. The bias of using S’ instead of Y in estimating ATE can be estimated by

$$\delta=\sum_{i=1}^{n} R_{i}\{\phi(Y_{i},A_{i},X_{i})-\phi(S_{i}',A_{i},X_{i})\}/\sum_{i=1}^{n} R_{i}.$$

Then, the calibrated estimation of ATE is the doubly robust estimator corrected by the estimated bias

$$\Delta=n^{-1}\sum_{i=1}^{n} \phi(S_{i}',A_{i},X_{i}) + \delta.$$

Next, we present the details on the method proposed by Cheng et al. [32] with specifics needed for implementation. The key is to construct S’ which can then be used to obtain an unbiased and doubly robust ATE estimator, employing a generalized g-formula for longitudinal studies [48].

Their method involves the following steps:

1. **Fitting a Double-Index Propensity Score (PS) Model:** To ensure robust to inadequate estimation of either PS or outcome regression (OR) model, Cheng et al. recommend using a double-index PS model that stratifies the observations along two dimensions including both the estimated PS and OR. Such stratification will eliminate confounding within each stratum whenever one dimension reflects the true PS or OR model. The process begins with estimating an initial PS model using baseline confounders which provides an estimate for P(A=1|X). Next, an initial outcome model for E(Y|X,A) is estimated using gold-standard outcomes, baseline confounders and the treatment assignment indicator over labeled subset. The initial PS estimate is then refined to obtain the final PS, $\pi(X)$, via nonparametric kernel smoothing, regressing A against the two scores: one from the initial PS model, and the other from the outcome model.
2. **Outcome Calibration:** The calibration of imputed outcomes S involves refitting the outcome Y against S, A, and an augmentation inverse-probability-weighting (IPW) factor U = I(A = 1)/$\pi(X)$ - I(A=0)/{1-$\pi(X)$} as covariates. This calibration step ensures that the imputation error from calibrated imputation Y-S’ is asymptotically uncorrelated with the IPW factor. As the result, the IPW of gold-standard outcome Y is asymptotically equivalent to the IPW of calibrated imputation S’, $E[$YU] = $E[$S’U], removing potential bias in the ATE estimate due to misclassification of S for Y.
3. **ATE Estimation:** The ATE is estimated by IPW of calibrated imputation using double index PS $n^{-1}\sum_{i=1}^{n} S'_{i}U_{i}=n^{-1}\sum_{i=1}^{n} S'_{i}A_{i}/\pi(X_{i})-S'_{i}{(1-A}_{i})/\{1-\pi(X_{i})\}$.
